# IL-22 Signaling Contributes to West Nile Encephalitis Pathogenesis

**DOI:** 10.1371/journal.pone.0044153

**Published:** 2012-08-28

**Authors:** Penghua Wang, Fengwei Bai, Lauren A. Zenewicz, Jianfeng Dai, David Gate, Gong Cheng, Long Yang, Feng Qian, Xiaoling Yuan, Ruth R. Montgomery, Richard A. Flavell, Terrence Town, Erol Fikrig

**Affiliations:** 1 Section of Infectious Diseases, Department of Internal Medicine, Yale University School of Medicine, New Haven, Connecticut, United States of America; 2 Section of Rheumatology, Department of Internal Medicine, Yale University School of Medicine, New Haven, Connecticut, United States of America; 3 Department of Immunobiology, Yale University School of Medicine, New Haven, Connecticut, United States of America; 4 Regenerative Medicine Institute, Departments of Biomedical Sciences and Neurosurgery, Cedars-Sinai Medical Center, Los Angeles, California, United States of America; 5 Department of Medicine, University of California Los Angeles, Los Angeles, California, United States of America; 6 Howard Hughes Medical Institute, Chevy Chase, Maryland, United States of America; 7 Department of Biological Sciences, University of Southern Mississippi, Hattiesburg, Mississippi, United States of America; Blood Systems Research Institute, United States of America

## Abstract

The Th17 cytokine, IL-22, regulates host immune responses to extracellular pathogens. Whether IL-22 plays a role in viral infection, however, is poorly understood. We report here that *Il22^−/−^* mice were more resistant to lethal West Nile virus (WNV) encephalitis, but had similar viral loads in the periphery compared to wild type (WT) mice. Viral loads, leukocyte infiltrates, proinflammatory cytokines and apoptotic cells in the central nervous system (CNS) of *Il22^−/−^* mice were also strikingly reduced. Further examination showed that Cxcr2, a chemokine receptor that plays a non-redundant role in mediating neutrophil migration, was significantly reduced in *Il22^−/−^* compared to WT leukocytes. Expression of Cxcr2 ligands, *cxcl1 and cxcl5*, was lower in *Il22^−/−^* brains than wild type mice. Correspondingly, neutrophil migration from the blood into the brain was attenuated following lethal WNV infection of *Il22^−/−^* mice. Our results suggest that IL-22 signaling exacerbates lethal WNV encephalitis likely by promoting WNV neuroinvasion.

## Introduction

IL-22 is a member of the IL-10-related cytokine family [1], and has been implicated in both chronic inflammatory diseases and infectious diseases. The tissue-modulating function of IL-22 in response to the immune system sets it apart from IL-10, which regulates immune cell functions [2], [3]. Although known as a Th17 cytokine, IL-22 is also expressed by a wide range of immune cells, including NK T, γδ T, and NK cells [4]–[6]. However, its receptor is exclusively produced by tissue cells, including epithelial and endothelial cells [7], [8]. Activation of IL-22 receptor leads to Stat3, Stat1, MAPK kinase and Akt signaling, which then results in diverse outcomes such as cell proliferation and survival [9]–[13].

The role of IL-22 in inflammatory and infectious diseases varies with tissue and disease conditions. IL-22 contributes to pathogenesis of psoriasis by inducing the proinflammatory S100 family of calcium binding proteins [5], [7] and plays a role in multiple sclerosis by promoting leukocyte infiltration into the brain [14]. However, IL-22 protects the liver from immune system-mediated damage during hepatitis [12], [13]. Upon microbe assaults, especially extracellular pathogens such as *K. pneumonia* the host increases IL-22 expression, which helps maintain epithelial barriers and induces secretion of anti-microbial peptides by the epithelia [15]. Although IL-22 is also induced by virus [16], [17] and has been implicated in anti-HIV function [18], its *in vivo* role in viral infections has yet to be defined.

Mosquito-borne viruses in the *Flaviviridae* family have recently emerged as a threat to human health. One of the life-threatening flaviviruses, West Nile virus (WNV), is maintained worldwide in an enzootic cycle between avian hosts and mosquito vectors. Since the outbreak in New York City in 1999, WNV has been rapidly spreading throughout North America and has caused thousands of deaths in the U.S.A. Infection is usually asymptomatic in most cases, but elicits fever, meningitis, encephalitis or acute flaccid paralysis in 20–40% of individuals [19].

The mechanisms by which neurotropic flaviviruses including WNV enter the central nervous system (CNS) are not well understood. In theory, WNV can enter the brain through many routes including 1) endothelial tight junctions [20]–[26], 2) direct infection of endothelial cells [23], 3) infected leukocytes that traffic to the CNS [26], 4) infection of olfactory neurons [26] and 5) direct axonal retrograde transport from infected peripheral neurons [27]. Although infiltrating CD8 T cells are crucial for controlling WNV dissemination in the CNS [28]–[31], they are recruited to the CNS too late after viral invasion [day 6 post-infection (p.i.) in mouse models] to prevent initial infection [32]. Early entry of neutrophils (day 4 p.i. in the mouse) that carry a heavy load of neurotropic virus may be deleterious to host [33] and contribute to CNS inflammation [34].

Here, we studied the *in vivo* role of IL-22 in WNV infection and found that IL-22 contributed to the early entry of neutrophils into the CNS. Results show that IL-22 signaling contributes to WNV pathogenesis in the CNS.

## Materials and Methods

### Animals and Infection

All animals were housed in our state-of-the-art animal facility at Yale University. Age and sex-matched animals (5–8 weeks old C57BL/6 and *II22^−/−^*) were infected with WNV at a sublethal dose of 20 plaque forming units (PFU) via an intracranial (i.c) route, or 200 PFU via footpad subcutaneous (s.c) route, and mice were maintained in a BSL3 animal facility. *Il22^−/−^* mice have been described previously [13]. WNV (strain CT2741) was isolated from naturally-infected wild birds and *in vitro* passaged once in Vero cells. Animals were thoroughly perfused with 50 ml of 1x ice-cold phosphate buffered saline (PBS) prior to tissue analyses. All animal protocols were approved by the Yale University Institutional Animal Care & Use Committee (protocol 10404).

### Chemicals and Antibodies

Anti-IL-22Rα1, Cxcr2-Phycoerythrin, mouse Cxcl1 and IL-22 ELISA kits were purchased from R&D Systems (# MAB4294, FAB2164P, DY453, M2200), Anti-CD45 was from Serotec (1∶200; clone IBL-3/16; used as a pan-leukocyte marker), and anti-mouse neutrophils was from Cedarlane Labs (1∶200; clone 7/4). Mouse anti-WNV was a kind gift from Dr. John F. Anderson at the Connecticut Agricultural Experimental Station, New Haven, CT. Recombinant human IL-22 was purchased from PROSPEC (Ness Ziona, Israel), allophycocyanin (APC)-labeled anti-Ly6G (Gr-1) and fluorescein (FITC)-labeled anti-CD45 from BD Pharmingen (# 553129 and 553080).

### Cell Culture

Human PMNs were isolated from freshly-drawn blood according to a previous study [33]. PMNs were infected with WNV at a multiplicity of infection of 1 (MOI = 1) for 24 hours. Primary human brain microvascular endothelial cells (HBVECs) (ScienCell, #1000) were cultured in formulated ECM medium (ScienCell, #1001) and treated with human recombinant IL-22 at a concentration of 200 ng/ml. Total RNAs were isolated after infection/treatment, and then reverse-transcribed into cDNA using the BioRad iScript cDNA synthesis kit.

### Polymorphonuclear Leukocyte (PMN) Transendothelial Migration Assay

3×10^4^ HBVECs were grown in cell culture inserts (pore size 3.0 micron) coated with human fibronectin (BD Biosciences # 354543) for 3 days, allowing formation of a monolayer [14]. We assured integrity of the HBVEC layer by assessing Evans blue leakage. IL-22 was added to the wells of a culture plate (lower chamber) and 24 hours later the inserts was washed with PBS twice gently. 2×10^5^ PMNs in 0.5 ml medium were added to the inserts and the live cells in the lower chamber (1.0 ml medium) were counted after 18 h. We counted 4 times and took the average for each well. Primary mouse brain vascular endothelial cell (MBVEC) monolayer [35] were pre-treated with 1 µg of purified rat IgG or rat anti-mouse IL-22R (R&D # MAB4294) for 24 hours followed by 24 h-treatment with 200 ng/ml of mouse IL-22. 2.5×10^5^ purified mouse blood PMNs were added to the upper chamber and the numbers of PMNs that migrated out of the upper chamber across the MBVEC monolayer down to the lower chamber were counted after 18 hours.

### *Fluorescence-activated Cell Scanning (FACS) Analyses* of Whole Blood Cells and Brain Leukocytes

Mice were infected with 200PFU of WNV via s.c. footpad. At day 4 p.i., 200 µl of whole blood was collected for immunostaining. Briefly, 5 µg of mouse IgGs was added to the blood samples, which was then incubated at room temperature for10 min. 0.25 µg of FITC-labeled anti-CD45, APC-labeled anti-Ly6G (Gr-1) and PE-labeled anti-Cxcr2 was added to the blood, which was then incubated at 4°for 40 min. Cells were then pelleted, washed, fixed and analyzed by FACS using LSR II Flow cytometer (BD Bioscience). The mean fluorescence intensity of Cxcr2 on CD45^+^Ly6G^+^Cxcr2^+^ cells was quantified using Flowjo software. Brain leukocytes were isolated as described previously [32].

### Microscopy and Tunel Staining

Mouse tissues were fixed in 4% paraformaldehyde (PFA) and sectioned in paraffin. Immunofluorescence staining was performed following published standard protocols [36]. Terminal deoxynucleotidyl transferase dUTP nick end labeling (TUNEL) staining was carried out using a kit from Promega Corporation. Using this procedure, apoptotic nuclei are stained dark brown, and sections were counter-stained with hematoxylin (blue color). TUNEL images were acquired using a Zeiss AxioImager Z1 microscope. Sections reacted with anti-WNV antibody in presence of CD45 or 7/4 antibodies were incubated with appropriate secondary antibodies conjugated with Alexa Fluor 488 and Alexa Fluor 594 antibodies. The resulting sections were mounted in ProLong® Gold fluorescence mounting media containing DAPI (as a nuclear counter-stain) and imaged in independent channels using a confocal microscope (Nikon C1 Eclipse) according to our previously published methods [26].

### Quantitative RT-PCR (qPCR)

Taqman qPCR was performed using gene-specific primers and 6FAM-TAMRA probes [36] or inventoried gene expression primer-probe sets from Applied Biosystems (6FAM-MGB probes).

The primer sets for SYBRGreen PCR of human genes were: *CXCR2*, forward 5-CCGGGCGTGGTGGTGAG and reverse 5-AGACAGAGTCTCACTGTCG; *CXCR4*, forward 5-TCGTCCACGCCACCAACAGT and reverse 5-CTGTCATCTGCCTCACTG; *CXCL1*, forward 5-GCTTGCCTCAATCCTGCA and reverse 5-CAATCCAGGTGGCCTCTGC; *CXCL2*, forward: 5-GCTGCTGCTCCTGCTCCTG and reverse 5-CTTCGGTTTGGGCGCAGTG; *CXCL5*, forward 5-AAGGTGGCCACGCTGGGGCAA and reverse 5-TGCAACGTCAGGCAGTTGCCCT.

### Graphs and Statistics

Survival curves, charts and statistical analysis were performed using PRISM 4 software (Graphpad Software, San Diego, CA). Significance was assessed using unpaired two-tailed Students’ *t*-test or nonparametric Mann-Whitney U test. For all analyses, a cutoff p value of 0.05 was used.

## Results

### IL-22 Deficiency Confers Resistance to Lethal WNV Infection

**Figure 1:**
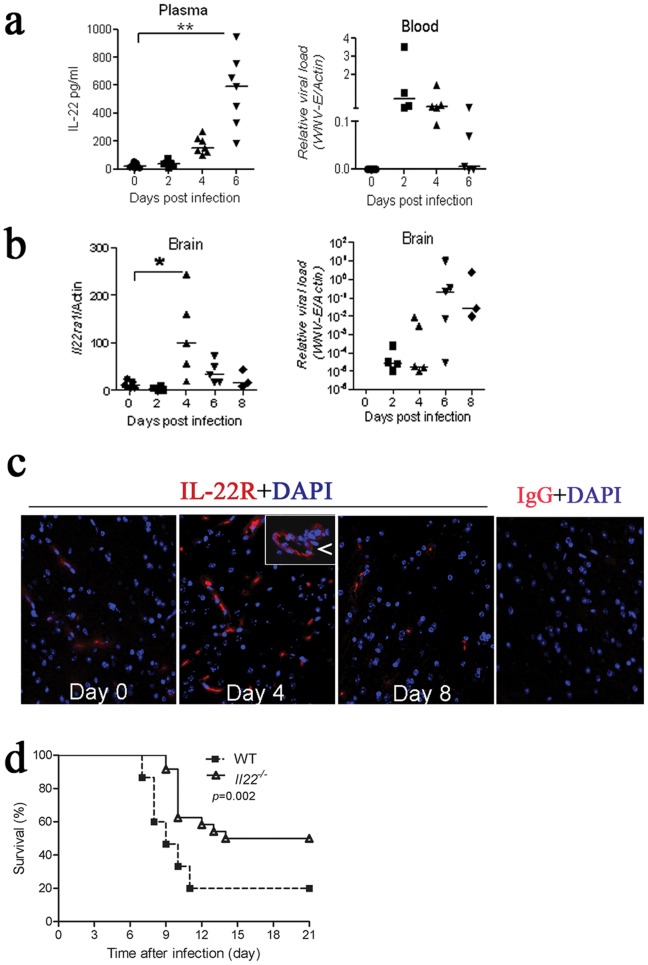
Il22^−/−^ mice are resistant to lethal WNV infection. **a**) Plasma IL-22 levels of WT mice were measured by ELISA, and viremia was quantified by q-PCR using WNV envelope gene (*WNV-E*) primers and probes and normalized with mouse actin. **b**) IL-22Rα1 transcripts in the WT mouse brain were examined by q-PCR and normalized with actin, and viral loads were quantified by q-PCR for *WNV-E* and normalized with mouse actin; *p<0.05; **p<0.01. Each dot represents one mouse. **c**) Immunofluorescence microscopy of IL-22Rα1 (red signal) in the WT mouse brain (cerebral cortex is shown). DAPI nuclear counter-stain is shown in blue. Arrows indicate blood vessels, shown in higher-magnification insets. **d**) Survival curve of *Il22* ^−/−^ *vs*. WT mice challenged with WNV. Mice were infected with 200 PFU of WNV via s.c. footpad injection and monitored daily for survival for 21 days (n = 30 for WT and n = 24 for *Il22* ^−/−^ mice). Data are pooled from 3 independent experiments.

IL-22 is expressed by immune cells at a low constitutive level; however, it is dramatically up-regulated under certain disease conditions [3]. We first tested if WNV infection induced IL-22 expression. As shown in Figure 1A, circulating IL-22 protein abundance was consistently increased from days 4 through 6 p.i by 6–20 fold compared to day 0. Interestingly, IL-22 levels remained high even though viremia receded at day 6 p.i. (Fig. 1A). The IL-22 receptor consists of 2 subunits, IL-10Rβ and IL-22R. Unlike IL-10Rα that is expressed by immune cells, IL-22R is exclusively produced by tissue cells [7], [8]. Indeed, we failed to detect IL-22R expression in blood leukocytes by RT-PCR. The mRNA levels of IL-22R in the brain, however, were readily detectable and increased by 5–10 fold at day 4 p.i. compared to day 0. Interestingly, IL-22R expression in the brain decreased at later time-points p.i. despite increasing viral loads (Fig. 1B, 1C). We then examined the survival response of IL-22 knockout mice (*Il22^−/−^*) to WNV challenge. Surprisingly, in contrast to its protective role in bacterial infection, IL-22 was detrimental to mice when challenged with WNV. Specifically, *Il22^−/−^* mice survived lethal WNV infection better than their wild-type (WT) counterparts (median survival 17.5 versus 9 days, *p*<0.01), and began to die on average, 2 days later (Fig. 1D).

### Central but not Peripheral Control of West Nile Virus in Il22^−/−^ Mice

**Figure 2:**
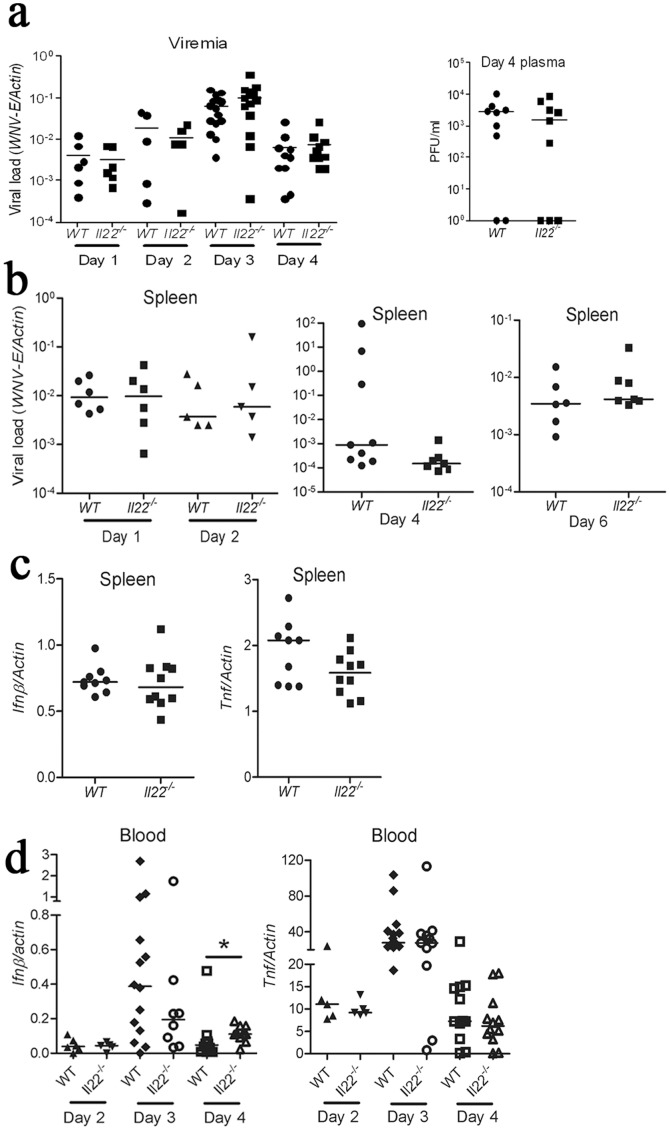
Phenotypic characterization of Il22^−/−^ mice after WNV challenge. Mice were infected with 200 PFU of WNV via s.c. footpad injection. Viral loads (shown by *WNV-E* expression and plaques per mg proteins) and cytokine/chemokine transcripts were quantified by q-PCR and normalized with mouse actin. Viral loads are shown from **a**) whole blood cells, **b**) spleen at the indicated days p.i. Cytokine transcripts are shown from **c**) spleen at day 4 p.i., **d**) in blood cells at the indicated days p.i. *p<0.05; **p<0.01. Each dot represents one mouse, and the horizontal lines are the medians of the results.

IL-22 signaling has been shown to control bacterial infection by helping to mount antimicrobial immune responses [6], [15]. We asked if IL-22 was important for controlling WNV dissemination and mobilizing peripheral anti-viral immune responses. There were no differences in viral loads in the blood or spleen between *Il22^−/−^* and wild type mice from day 1 through day 6 p.i. (Fig. 2A, 2B). Furthermore, *ifnb* and *tnfa* transcripts in spleens of *Il22^−/−^* mice remained largely similar to those of WT mice (Fig. 2C). Consistently, no significant differences in *tnfa* transcripts were observed in whole blood (Fig. 2D). These data indicate that IL-22 does not influence WNV infectivity and plays a sub-dominant role in these cytokine responses in the periphery.

**Figure 3:**
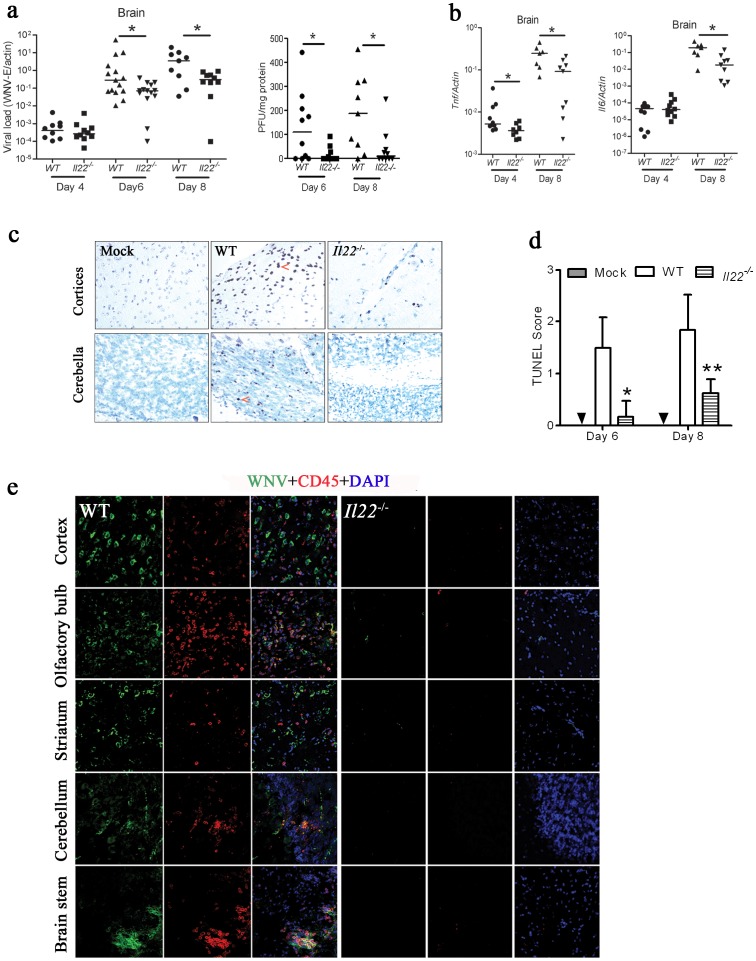
Phenotypic characterization of Il22^−/−^ mice after WNV challenge. Mice were infected with 200 PFU of WNV via s.c. footpad injection. On days4, 6 and 8 p.i., **a**) viral loads (shown by *WNV-E* expression and plaques per mg proteins) and **b**) cytokine transcripts from the brain were quantified by q-PCR and normalized with mouse actin. **c**) Paraffin embedded brain sections were reacted with TUNEL. Graphs indicate cerebral cortices representative of 5 mice per group. **d**) TUNEL scores were assessed using a semi-quantitative scale (from 0–3, with 3 indicating the greatest number of TUNEL positive cells); *p<0.05; **p<0.01, and the arrows indicate apoptotic cells. **e**) Cryosectioned brain samples from day 6 p.i. were stained for WNV envelope protein (green), CD45 (as a pan-leukocyte marker; red), and DAPI (blue) and utilized for laser scanning confocal microscopy (20X images are shown and are representative of n = 5 mice per group).

We then next examined WNV burden and cytokine expression in the CNS. Consistent with the survival result (Fig. 1D), CNS viral loads were significantly reduced in *Il22* ^−/−^ mice compared with WT at days 6 and 8 p.i. (Fig. 3A). The expression kinetics of select cytokines in the CNS of WT mice following WNV infection was first determined, with the highest induction noted at day 8 p.i compared to day 0 (Fig. S1). The expression levels of inflammatory cytokines, *tnfa* and *il6*, were decreased at day 8 p.i. in *Il22* ^−/−^ when compared to WT (Fig. 3B). We next examined histopathology in brain tissues of WT and *Il22* ^−/−^ mice. Consistent with the viral load results, TUNEL positive cells in *Il22* ^−/−^ brains were fewer in number than WT mice (Day 6, 182±21 v.s 28±4, per field under 10× microscope; day 8, 241±27 v.s. 102±13. p<0.05). A similar pattern of results was detected in multiple brain regions, including the cerebral cortex (Fig. 3C), olfactory bulb, striatum, cerebellum, and brainstem. As shown in Figure 3D, the TUNEL scores of *Il22* ^−/−^ mice were significantly lower than WT mice on days 6 and 8 p.i.(Fig. 3D). Leukocyte infiltration into the CNS is a hallmark of WNV encephalitis, and we observe this phenotype in mouse models of lethal WNV encephalitis [29]. Indeed, CD45^+^ leukocytes/microglia were readily detected in various brain regions of WT mice, particularly in areas earmarked by WNV antigen expression. However, the number of CD45^+^ cells was greatly reduced in *Il22* ^−/−^ mouse brain regions at both days 6 and 8 p.i.(Fig. 3E, Fig. S2).

### IL-22 Facilitates WNV Entry into the CNS

**Figure 4:**
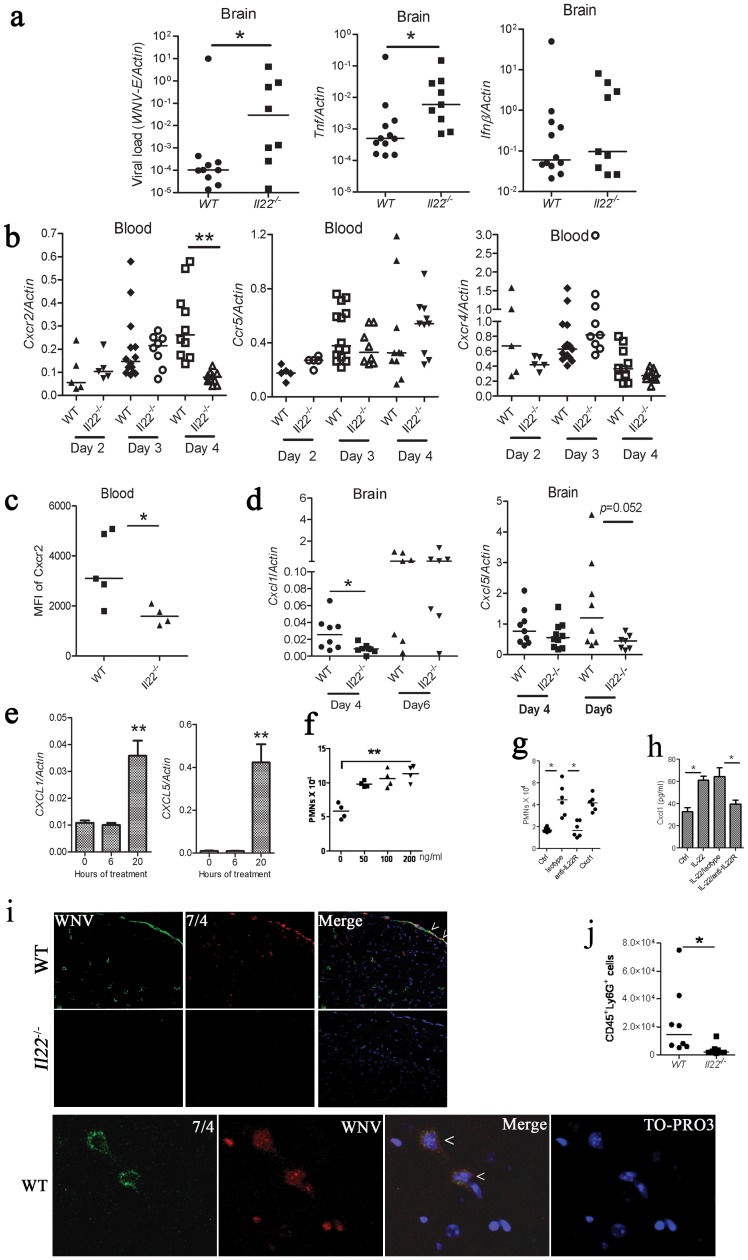
Brain neutrophil migration is defective in Il22^−/−^mice after WNV infection. **a**) 20 PFU of WNV was inoculated intracranially. At day 5 p.i., brain viral loads (shown by *WNV-E* expression) and cytokine/chemokine or chemokine receptor transcripts were quantified by q-PCR and normalized with mouse actin. **b–d, f**) Mice were infected with 200 PFU of WNV via s.c. footpad injection. **b**) Expression of select chemokine receptors by blood cells was quantified by q-PCR using specific primers and probes, and normalized with beta actin. **c**) The Cxcr2 expression levels of whole blood cells at day 4 p.i. were determined by immunostaining and flow cytometry. The results are expressed as mean fluorescence intensity (MFI) per cell. **d**) The mRNA levels of *cxcl1* and *cxcl5* in WT and *Il22* ^−/−^ brains were assessed by q-PCR. Each dot in the plot represents one mouse; *p<0.05; **p<0.01. **e**) Expression of *CXCL1* and *CXCL5* mRNA by human brain microvascular endothelial cells (HBVEC) treated with human recombinant IL-22. Bars: the mean of the results with standard deviation. **p<0.01 (*n* = 3). **f**) Numbers of human PMNs that migrated out of the upper chamber (2×10^5^) across the HBVEC monolayer down to the lower chamber over 18 hours in the presence of various concentrations of IL-22. Each dot represents the mean cell number from one well (counted 4 times). **p<0.001. **g**) Numbers of mouse PMNs that migrated out of the upper chamber (2×10^5^) across the mouse brain vascular endothelial cell (MBVEC) monolayer down to the lower chamber. Each dot represents the mean cell number from one well (counted 4 times). *p<0.05. **h**) Quantification of Cxcl1 levels of MBVECs treated as in **g**) by ELISA. **i**) Immunofluorescence staining of WNV-E (green), neutrophils (7/4, red) and DAPI (blue) in the brain cortex sections at day 5 p.i. Arrows point to co-staining. Micrographs were acquired using a laser scanning confocal microscope (20X images are shown and are representative of n = 5 mice per group). A higher magnification (63x) of confocal images of WT brain section is shown below (WNV, red; 7/4, green, TO-PRO3, blue). Arrow heads indicate typical polynuclear morphology of neutrophils. **j**) FACS analyses of brain neutrophils (CD45Ly6G^+^) of WT and *Il22* ^−/−^ mice on day 6 p.i. Each dot represents 2 mice. *p<0.05.

Since *Il22* ^−/−^ mice had lower viral loads than WT mice in the CNS (Fig. 3A) but not in the periphery (Fig. 2A), we hypothesized that IL-22 facilitates virus entry into the CNS. To exclude the possibility that IL-22 deficiency favored viral clearance in the brain, we performed intracranial inoculation with a low dose of WNV. Surprisingly, viral loads in the brain of *Il22* ^−/−^ mice were much higher than those in WT animals. Consistently, expression of the proinflammatory cytokine TNF was also significantly increased (Fig. 4A). These results, nevertheless, disproved the possibility that IL-22 deficiency favored viral clearance in the CNS when virus was inoculated via s.c route, and suggested that IL-22 signaling facilities WNV entry to the CNS.

Neutrophils are the predominant cell infiltrate in cerebrospinal fluid from patients with WNV meningitis and encephalitis [37], [38]. These cells have been suggested to be a key reservoir for WNV in the blood, and deletion of Cxcr2, a chemokine receptor that mediates neutrophil migration, alleviated WNV infection of the CNS in a mouse model [33]. We next asked whether IL-22 impacted Cxcr2 expression and regulated CNS infiltration of neutrophils. As shown in Figure 4B, *cxcr2* mRNA expression by blood leukocytes was significantly lower in *Il22* ^−/−^ than that in WT mice at day 4 p.i., a time when neutrophils begin penetrating the CNS in our peripheral murine WNV infection model. This effect was specific to *cxcr2*; since expression of the chemokine receptors *ccr5* and *cxcr4* remained similar between WT and *Il22* ^−/−^ mouse peripheral blood cells (Fig. 4B). To determine whether the population of Cxcr2-positive blood cells or average Cxcr2 expression level was affected in *Il22^−/−^*, we then analyzed the numbers and mean fluorescence intensity of CD45 (pan leukocytes) Ly6G (granulocytes and monocytes) Cxcr2-positive cells in the blood. At day 4 p.i., the numbers of this population were similar between WT and *Il22^−/−^*, but the MFI of Cxcr2 was much lower in *Il22^−/−^* (Fig. 4C, Fig. S3), suggesting that IL-22 contributes to Cxcr2 expression. Cxcl1 and Cxcl5, Cxcr2 ligands that are expressed also by brain cells, stimulate chemotaxis of neutrophils by interacting with Cxcr2. We further examined the abundance of *cxcl1* and *cxcl5* transcripts in brains of WT and *Il22^−/−^*. At day 4 and 6 p.i., *cxcl1* and *cxcl5* transcripts were decreased by two-fold respectively in *Il22* ^−/−^ brains compared to WT (Fig. 4D), suggesting that IL-22 contributes to Cxcl1 and Cxcl5 expression. The brain microvascular endothelial cells (BVECs) regulate leukocyte traffic in part by producing a chemokine gradient. We reasoned that IL-22 impacts chemoattractant expression by BVECs and neutrophil migration across endothelia. Indeed, human primary BVECs (HBVECs) upregulated *CXCL1* and *CXCL5* mRNA expression by over 3- and 40-fold respectively when treated with recombinant IL-22 for 20 h (Fig. 4E). Similar results were observed with mouse BVECs (Fig. S4A, S4B). These data indicate that IL-22 aids in neutrophil recruitment by regulating chemoattractant production at the blood brain barrier (BBB). To further strengthen this point, we tested the ability of PMNs to cross a HBVEC monolayer in the presence of IL-22. Indeed, after HBVECs were treated with 50–200 ng/ml of IL-22 for 24 h, more PMNs were found in the lower chamber than the control (0 ng/ml) (Fig. 4F). We next employed mouse MBVECs to examine the impact of IL-22 signaling on mouse PMN migration. Similar results were observed (Fig. 4G). Furthermore blocking IL-22 signaling using a monoclonal anti-IL-22R reduced PMN transmigration (Fig. 4G, 4H). In addition, in response to Cxcl1 the neutrophils from WNV-infected *Il22* ^−/−^ mice transmigrated a MBVEC layer less efficiently than those from WT (Fig. S4C). *In vivo*, at day 6 p.i., both WNV and neutrophils were present in the WT, but nearly absent in *Il22* ^−/−^ brains (Fig. 4I). This was further confirmed by FACS analyses of brain neutrophils (CD45Ly6G positive) (Fig. 4J). Interestingly, neutrophils in WT mouse brains co-localized with WNV in the fairly vascularized meninges (Fig. 4I), further suggesting that neutrophils may act as CNS virus carriers.

## Discussion

The roles of IL-22 in inflammation and bacterial infections are diverse [39]. However, its *in vivo* function in viral infections has yet been defined. Our results support a novel concept that IL-22 regulates anti-viral responses, likely by refereeing neutrophil infiltration of the CNS. Unlike *Il10* deficiency or *Tlr7* sufficiency, which leads to enhanced innate immune responses and better control of WNV infection *in vivo* [40], [41], deletion of IL-22 had no significant impact on innate immune responses to WNV. Rather, *Il22* ^−/−^ mice demonstrated more resilience to s.c. lethal WNV infection and reduced virus entry into the CNS.

Previous studies have shown that both IL-17 and IL-22 contribute to multiple sclerosis lesions by promoting transmigration of Th17 lymphocytes across the blood brain barrier [14]. Our results extend this idea into neutrophil biology, and suggest that IL-22 facilitates neutrophil infiltration of the CNS after WNV infection. Our statement is supported by several lines of evidence. First, IL-22 signaling sustains expression of Cxcr2, a neutrophil receptor that plays a critical role in chemotaxis of these cells, after WNV challenge. WT animals upon WNV challenge increased the serum levels of IL-22 and Cxcr2 expression consistently over the time of observation (day 2 through 4). *Il22* ^−/−^ mice increased Cxcr2 expression at day 3 (compared to day 2), but failed to maintain high levels of Cxcr2 at day 4 p.i. These data suggest that IL-22 serves to sustain Cxcr2 expression after peripheral infection has been established. However, it is unlikely that IL-22 directly acts on neutrophils to upregulate Cxcr2 expression as neutrophils do not express IL-22R. Rather, modulation of Cxcr2 by IL-22 may be indirect via IL-22-induced proinflammatory mediators such as TNF and IL-8 [42], potent inducers of Cxcr2. Second, IL-22 signaling can induce the expression of CXCR2 ligands, CXCL1 and CXCL5, in endothelial cells. In the absence of IL-22, *cxcl1* and *cxcl5* mRNA in mouse brains was reduced after WNV infection. The decrease in *cxcl1* appeared at day 4, while the difference in *cxcl5* was observed at day 6. This might due to the differential kinetics of gene expression during WNV infection. In agreement with a previous study [43], *in vivo cxcl1* was an early responsive gene and upregulated by WNV infection dramatically; while *cxcl5* was only modestly induced at a later time. Third, activation of IL-22 signaling in BVECs promotes neutrophil migration across BVEC monolayer. *In vitro*, treatment of BVECs with recombinant IL-22 allowed neutrophils to cross BVEC layer more efficiently. *In vivo*, IL-22 deficiency reduced the number of neutrophils in brain perivascular spaces and parenchyma after WNV infection. This is in agreement with a recent study showing that IL-22 induces transient elevation of circulating neutrophils via CXCL1 in the liver [44]. Taken all together, these data suggest that IL-22 favors neutrophil recruitment to the CNS by regulating CXCR2-CXCL1 axis.

Leukocyte infiltrates in the CNS may be favorable of virus clearance, but also neuron inflammation. Clearly, infiltrating CD8 T cells are crucial for controlling WNV dissemination in the CNS [28]–[31], but these cells also exacerbate CNS pathology induced by Japanese encephalitis virus, the closest relative of WNV in the *Flaviviridae* family [45]. Accumulation of monocytes may be harmful to mice infected with WNV [46]. However, Ccr2-mediated Ly6C^hi^monocytosis and accumulation in the brain is protective against WNV infection [47]. The most abundant leukocytes, neutrophils, are the predominant cell infiltrate in the cerebrospinal fluid from patients with WNV meningitis and encephalitis [37], [38], and in mice they are present in the brain parenchyma as early as day 4 p.i. [32]. These cells have also been demonstrated to support WNV infection very effectively [33]. It is thus plausible that WNV is carried by neutrophils to cross the blood brain barrier. Indeed, our data show that neutrophils in WT mouse brains co-localized with WNV in the vascularized meninges and depletion of neutrophils protected mice from lethal WNV infection [33]. Neutrophils in the CNS could also contribute to CNS inflammation [34] and BBB disruption by producing inflammatory mediators such as matrix metalloproteinases.

Similar to Cxcr2 knockout mice challenged with WNV [33], *Il22* ^−/−^ mice died 2 days later than WT, suggesting a delayed entry of virus into the CNS. The overall mortality of *cxcr2* ^−/−^ was as the same as WT, while *Il22* ^−/−^ survived better than WT. A possible explanation is that *Il22* ^−/−^ showed a decrease in Cxcr2 expression after the peripheral infection had established (day 4 p.i.), and *Il22* ^−/−^ were also defective in brain Cxcl1 and Cxcl5 expression. Thus IL-22 does not influence neutrophil function, either favoring WNV dissemination initially or then controlling WNV infection later. Rather IL-22 specifically regulates neutrophil migration and thus WNV entry into the CNS after peripheral infection has been established. Surprisingly though, IL-22 appeared beneficial to the host when virus was directly inoculated into the brain. This is in agreement with previous observations showing that IL-22 was implicated in the control of Theiler’s virus dissemination in the spinal cord when virus was inoculated intracerebrally [48]. It is likely that IL-22 produced by the CNS resident cells may restrict viral dissemination by inducing local immune response such as defensins [7], potent anti-viral peptides [49]. However, activation of IL-22 signaling could also have immunopathogenic effects, leading to exacerbated disease conditions [5], [24], [48]. Nonetheless, our results demonstrate that IL-22 signaling facilitates WNV pathogenesis in mice, likely by promoting infiltration of infected neutrophils from the blood into the brain.

## Supporting Information

Figure S1 **Expression kinetics of cytokines following WNV infection.** Mice were infected with 200 PFU of WNV via s.c. footpad injection. The mRNA levels of indicated cytokines from whole blood cells or brain cells were quantified by Taqman q-PCR and normalized with beta actin gene. Each dot represents one mouse and the horizontal line indicates the median of the results. Significant induction of cytokines in blood cells from days 1 through 5, and in the brain of day 8 are observed compared with uninfected (day 0).
(PDF)

Figure S2 **Reduced WNV histopathology in Il22^−/−^ brains.** Mice were infected with 200 PFU of WNV via s.c. footpad injection. At day 8 p.i., mice were euthanized and perfused. Cryosectioned brain samples from day 8 p.i. were stained for WNV envelope protein (green), CD45 (as a pan-leukocyte marker; red), and DAPI (blue) and utilized for laser scanning confocal microscopy (20X images are shown and are representative of n = 5 mice per group).
(PDF)

Figure S3 **FACS analysis of blood cells.** Mice were infected with 200 PFU of WNV via s.c. footpad injection. Blood cells at day 4 p.i. were stained for CD45-FITC, Ly6G-APC, and **a)** isotype control (rat IgG2A) or Cxcr2-PE **b)** from WT mice, or **c)** from *Il22* ^−/−^ mice.
(PDF)

Figure S4 **IL-22 signaling promotes PMNs migration.** **a)** Induction of *cxcl* by IL-22 in MBVEC. MBVECs were treated with 200 ng/ml of mouse IL-22 for the indicated time duration. The mRNA levels of *cxcl1* and *cxcl5* were quantified by quantitative RT-PCR and normalized with mouse beta actin gene. *p<0.05. **b)** Detection of MBVEC IL-22R by immunoblotting (IB). **c)** Migration of blood PMNs from WT or *Il22* ^−/−^ infected with WNV for 4 days (blood was pooled from 8 mice) across a MBVEC monolayer in the presence of 200 ng/ml mouse Cxcl1 after 24 h. Each dot represents the mean cell number from one well. *p<0.05.
(PDF)
