# Supplementary material for: IL-22 Signaling Contributes to West Nile Encephalitis Pathogenesis
Source: PLoS One. 2012 Aug 28;7(8):e44153. doi: 10.1371/journal.pone.0044153 (PMC3429482; doi:10.1371/journal.pone.0044153)
Supplement: Figure S1 — Expression kinetics of cytokines following WNV infection. Mice were infected with 200 PFU of WNV via s.c. footpad injection. The mRNA levels of indicated cytokines from whole blood cells or brain cells were quantified by Taqman q-PCR and normalized with beta actin gene. Each dot represents one mouse and the horizontal line indicates the median of the results. Significant induction of cytokines in blood cells from days 1 through 5, and in the brain of day 8 are observed compared with uninfected (day 0). (PDF) [file pone.0044153.s001.pdf]

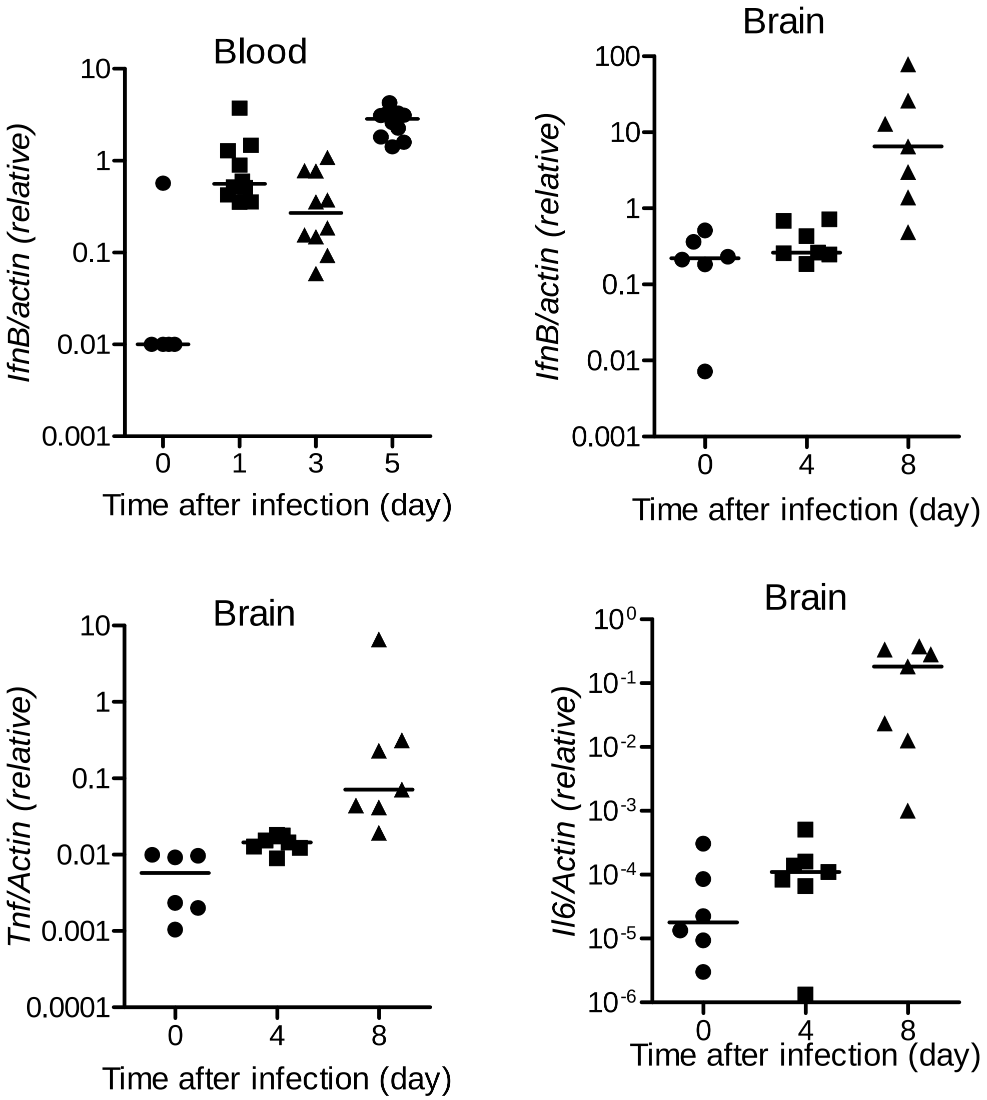

Figure S1. Expression kinetics of cytokines following WNV infection. Mice were infected with 200 PFU of WNV via s.c. footpad injection. The mRNA levels of indicated cytokines from whole blood cells or brain cells were quantified by Taqman q-PCR and normalized with beta actin gene. Each dot represents one mouse and the horizontal line indicates the median of the results. Significant induction of cytokines in blood cells from days 1 through 5, and in the brain of day 8 are observed compared with uninfected (day 0).
