# Supplementary material for: IL-22 Signaling Contributes to West Nile Encephalitis Pathogenesis
Source: PLoS One. 2012 Aug 28;7(8):e44153. doi: 10.1371/journal.pone.0044153 (PMC3429482; doi:10.1371/journal.pone.0044153)
Supplement: Figure S2 — Reduced WNV histopathology in Il22−/− brains. Mice were infected with 200 PFU of WNV via s.c. footpad injection. At day 8 p.i., mice were euthanized and perfused. Cryosectioned brain samples from day 8 p.i. were stained for WNV envelope protein (green), CD45 (as a pan-leukocyte marker; red), and DAPI (blue) and utilized for laser scanning confocal microscopy (20X images are shown and are representative of n = 5 mice per group). (PDF) [file pone.0044153.s002.pdf]

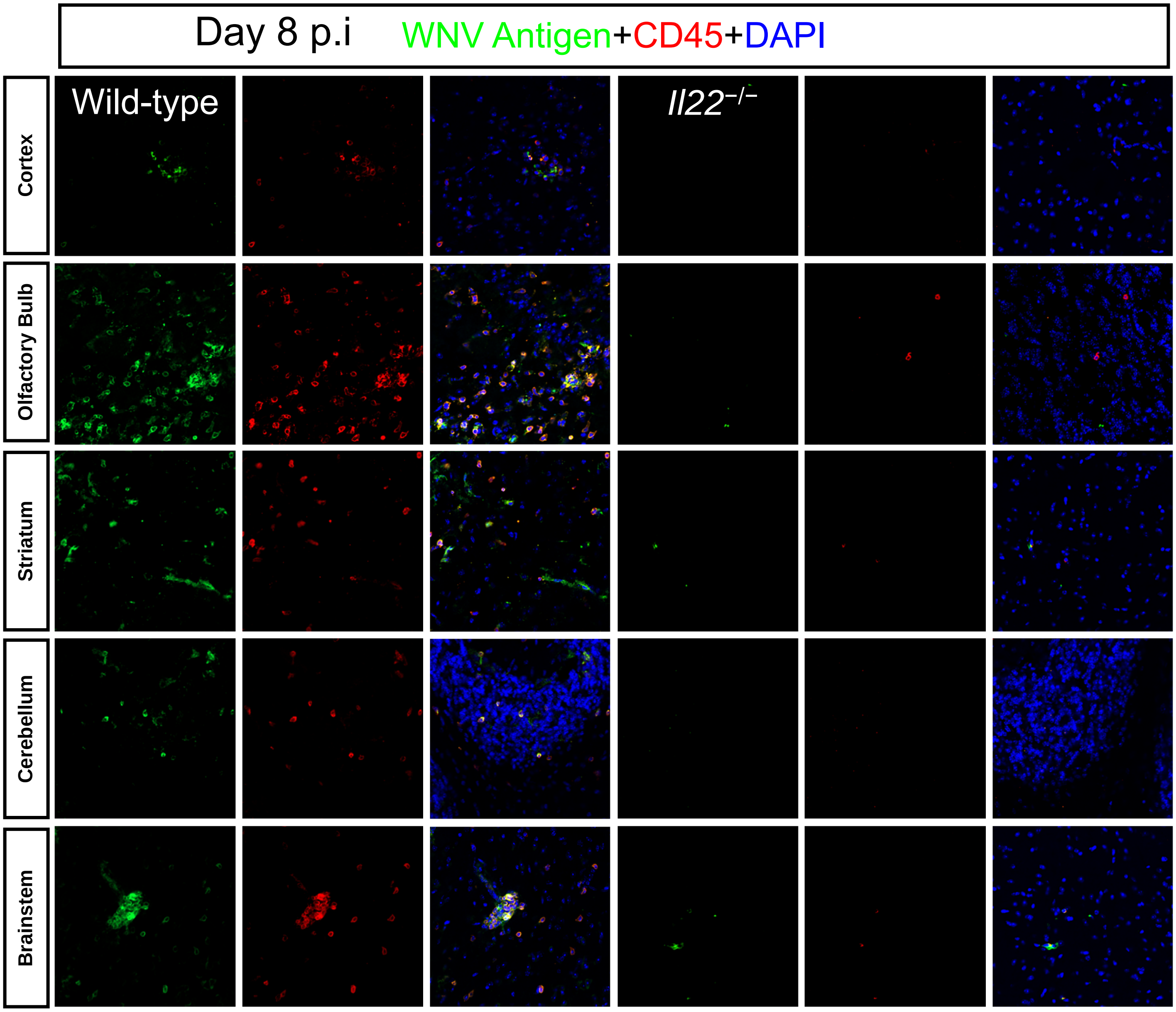

Figure S2. Reduced WNV histopathology in *Il22*$^{-/-}$ brains. Mice were infected with 200 PFU of WNV via s.c. footpad injection. At day 8 p.i., mice were euthanized and perfused. Cryosectioned brain samples from day 8 p.i. were stained for WNV envelope protein (green), CD45 (as a pan-leukocyte marker; red), and DAPI (blue) and utilized for laser scanning confocal microscopy (20X images are shown and are representative of n=5 mice per group).
