# Supplementary material for: IL-22 Signaling Contributes to West Nile Encephalitis Pathogenesis
Source: PLoS One. 2012 Aug 28;7(8):e44153. doi: 10.1371/journal.pone.0044153 (PMC3429482; doi:10.1371/journal.pone.0044153)
Supplement: Figure S3 — FACS analysis of blood cells. Mice were infected with 200 PFU of WNV via s.c. footpad injection. Blood cells at day 4 p.i. were stained for CD45-FITC, Ly6G-APC, and a) isotype control (rat IgG2A) or Cxcr2-PE b) from WT mice, or c) from Il22 −/− mice. (PDF) [file pone.0044153.s003.pdf]

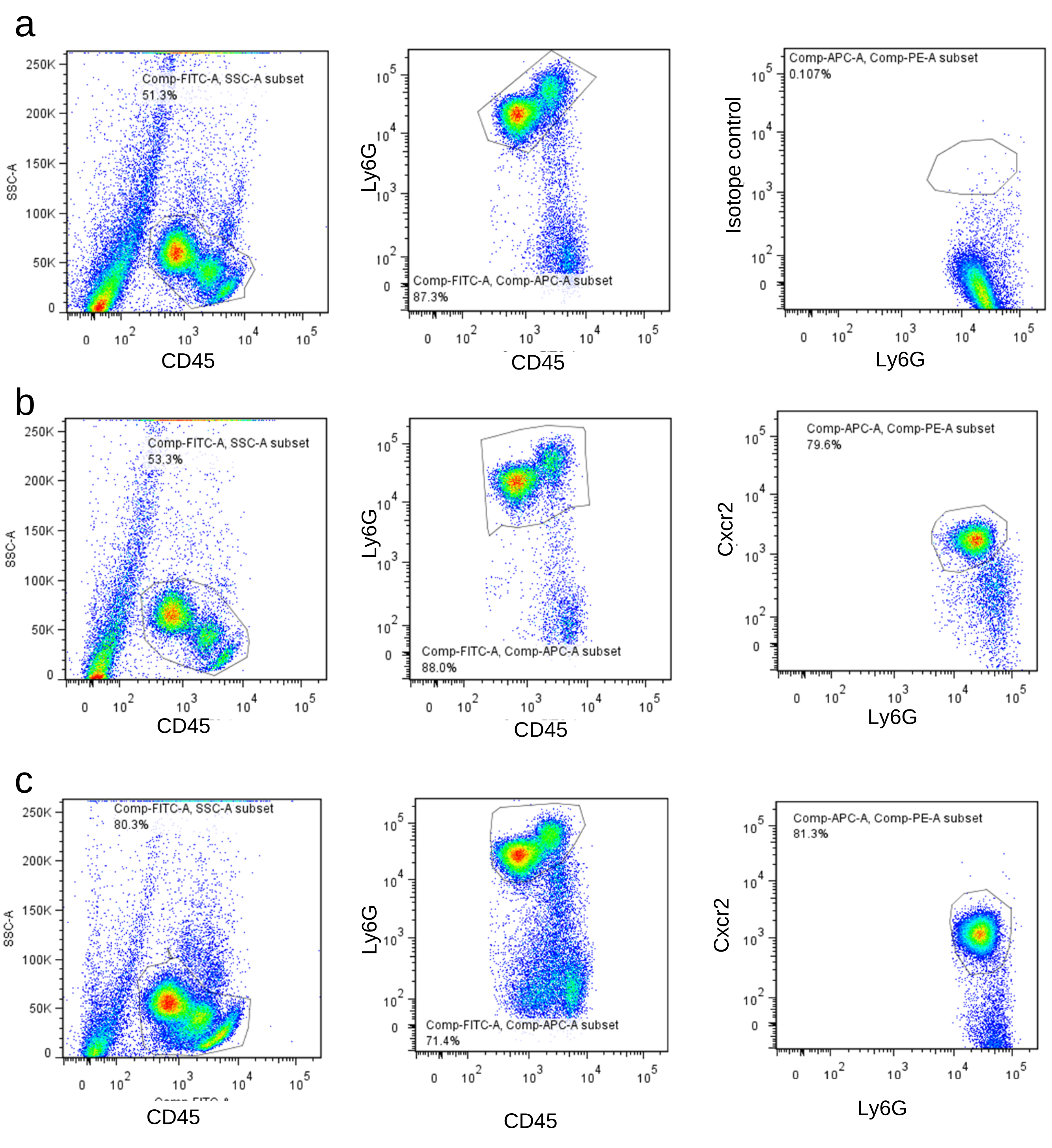

Figure S3. FACS analysis of blood cells. Mice were infected with 200 PFU of WNV via s.c. footpad injection. Blood cells at day 4 p.i. were stained for CD45-FITC, Ly6G-APC, and **a)** isotype control (rat IgG_{2A}) or Cxcr2-PE **b)** from WT mice, or **c)** from *Il22*$^{-/-}$ mice .
