# Supplementary material for: IL-22 Signaling Contributes to West Nile Encephalitis Pathogenesis
Source: PLoS One. 2012 Aug 28;7(8):e44153. doi: 10.1371/journal.pone.0044153 (PMC3429482; doi:10.1371/journal.pone.0044153)
Supplement: Figure S4 — IL-22 signaling promotes PMNs migration. a) Induction of cxcl by IL-22 in MBVEC. MBVECs were treated with 200 ng/ml of mouse IL-22 for the indicated time duration. The mRNA levels of cxcl1 and cxcl5 were quantified by quantitative RT-PCR and normalized with mouse beta actin gene. *p<0.05. b) Detection of MBVEC IL-22R by immunoblotting (IB). c) Migration of blood PMNs from WT or Il22 −/− infected with WNV for 4 days (blood was pooled from 8 mice) across a MBVEC monolayer in the presence of 200 ng/ml mouse Cxcl1 after 24 h. Each dot represents the mean cell number from one well. *p<0.05. (PDF) [file pone.0044153.s004.pdf]

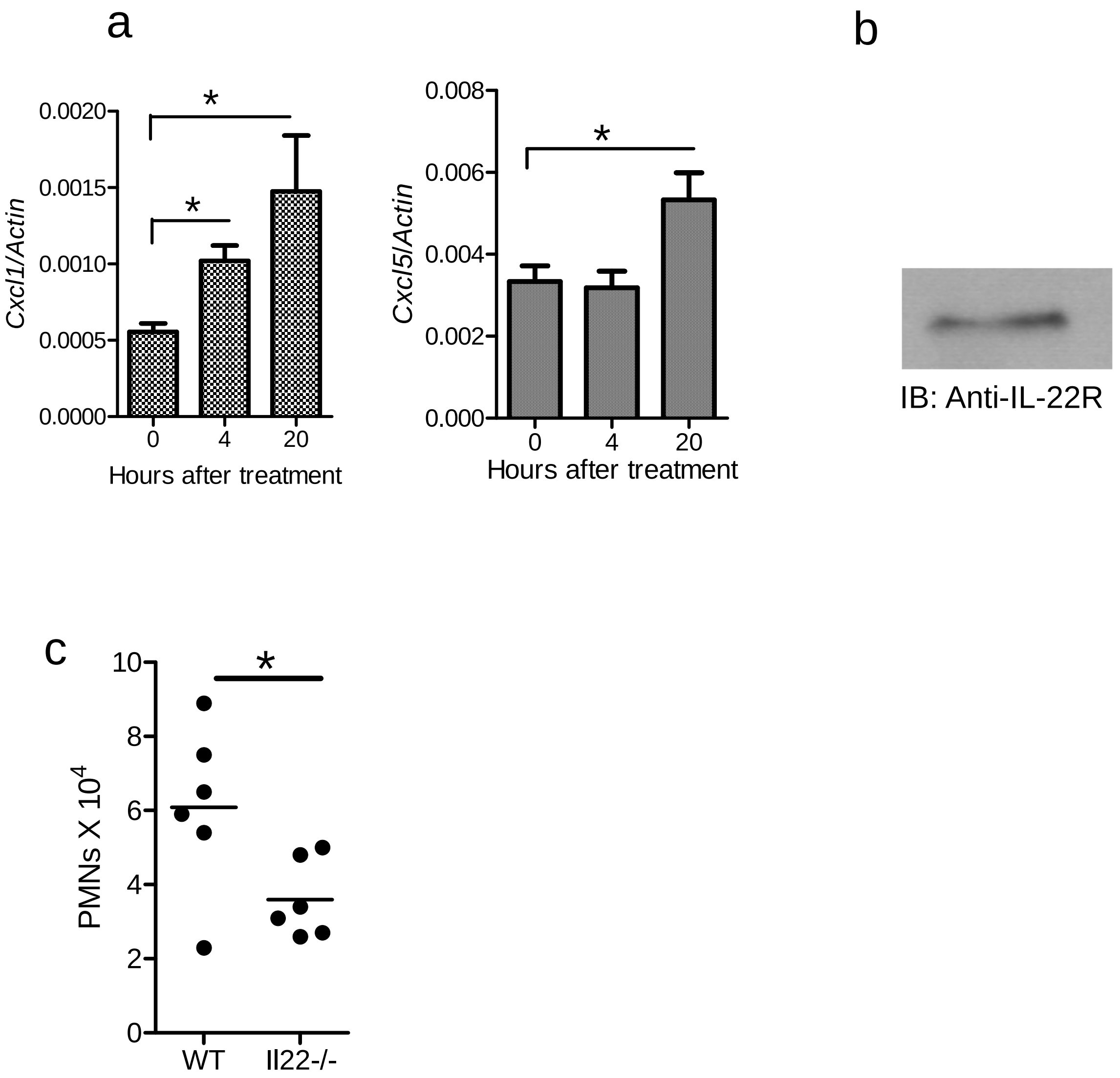

Figure S4. IL-22 signaling promotes PMNs migration. **a)** Induction of *cxcl* by IL-22 in MBVEC. MBVECs were treated with 200ng/ml of mouse IL-22 for the indicated time duration. The mRNA levels of *cxcl1* and *cxcl5* were quantified by quantitative RT-PCR and normalized with mouse beta actin gene. *, $p<0.05$. **b)** Detection of MBVEC IL-22R by immunoblotting (IB). **c)** Migration of blood PMNs from WT or *Il22*$^{-/-}$ infected with WNV for 4 days (blood was pooled from 8 mice) across a MBVEC monolayer in the presence of 200ng/ml mouse Cxcl1 after 24h. Each dot represents the mean cell number from one well. *, $p<0.05$.
